# Is There a Rationale for Haemoadsorption with Combined Use of CytoSorb^®^ and Oxiris^®^ in Patients with Underlying Viral Infection and Secondary Bacterial Sepsis?

**DOI:** 10.3390/jcm14196931

**Published:** 2025-09-30

**Authors:** Anna Wrzosek, Tomasz Drygalski, Łukasz Nowak, Izabella Grabowska, Jerzy Wordliczek, Michał Terlecki, Jarosław Garlicki

**Affiliations:** 1 Department of Interdisciplinary Intensive Care, Jagiellonian University Medical College, 30-688 Krakow, Poland; 2 Department of Anaesthesiology and Intensive Therapy, University Hospital in Kraków, 30-688 Krakow, Poland; 3 Centre for Extracorporeal Therapy, University Hospital in Kraków, 30-688 Krakow, Poland; 4 Department of Anaesthesiology and Intensive Therapy, Jagiellonian University Medical College, 31-501 Krakow, Poland

**Keywords:** sepsis, blood purification, haemoadsorption, Cytosorb^®^, Oxiris^®^, coronavirus, COVID-19, critical care, ICU

## Abstract

**Aim:** The rationale for combining various extracorporeal blood purification techniques to improve patient outcomes is currently being discussed extensively. The combined use of CytoSorb^®^, with high capacity for cytokine removal, and Oxiris^®^, which adsorbs endotoxins and smaller cytokines, may enhance the efficacy of blood purification in sepsis. **Study Design:** Retrospective analysis of efficacy and safety of simultaneous use of CytoSorb^®^ and Oxiris^®^ in 12 consecutive critically ill patients with COVID-19, who developed secondary bacterial sepsis and persistent hemodynamic instability. **Results:** Most of the patients (*n* = 8) treated with combination of the Oxiris^®^ and CytoSorb^®^ had a significant decrease in vasopressor requirement. Pre- and post-haemoadsorption data were analysed in 9 patients, who completed a 24 h course of treatment. A significant decrease in mean SOFA score (16.3 ± 1.7 to 15.0 ± 2.0 points), median vasopressor requirement (0.56 ± 0.29 to 0.11 ± 0.21 µg/kg/min), median procalcitonin levels (6.5 ± 27.0 to 1.6 ± 6.0 ng/mL), median IL-6 levels (584 ± 6279 to 107 ± 571 pg/mL), and mean leucocyte count (36.0 ± 20.6 to 20.9 ± 10.1 × 10^3^/mL) was observed. Furthermore, there was significant increase in PaO_2_/FiO_2_ ratio (108 ± 30 to 185 ± 55). We did not observe any device-associated adverse events or technical problems. A 27.5% drop in platelet count (269 ± 116 to 195 ± 82 × 10^6^/mL) and an 11.8% drop in haemoglobin level (10.7 ± 2.9 to 9.5 ± 2.0 g/dL) was noted. **Conclusions:** Our data suggests that combined use of Oxiris^®^ and CytoSorb^®^ for simultaneous cytokine and endotoxin removal in patients with underlying viral infection may be a promising therapeutic option. Our findings may serve as a guide for future research and provide directions for further development in this field.

## 1. Background

Coronavirus disease (COVID-19), caused by the SARS-CoV-2 virus, is a serious health condition that has had a significant impact on public health and caused millions of deaths worldwide. The main features of COVID-19 are uncontrolled systemic inflammation and coagulopathy, which, consequently, lead to severe complications and death [1]. The COVID-19 pandemic posed a huge challenge in the area of intensive care, and it was not uncommon to use unconventional therapies in critically ill patients. Now that the pandemic has subsided, it is worthwhile to analyse the various treatment strategies used during the pandemic. Such analysis may provide valuable material to assess the safety and efficacy of particular therapeutic interventions in other groups of patients (not only those with SARS-CoV-2 infection), e.g., patients with sepsis.

An important feature of SARS-CoV-2 infection was that patients with COVID-19 exhibited severe immunological dysregulation and disruption [2]. As a consequence, they were predisposed to secondary bacterial infections, which often led to an immune over-response to the original insult. This was associated with sustained excessive release of inflammatory mediators and manifested as fulminant septic shock with very high mortality and complication rates [3]. These observations underscored the need for adjuvant, inflammation-modulating therapies that could improve survival in this group of patients. One of the strategies explored was extracorporeal blood purification (EBP), including haemoadsorption and its various modalities. Haemoadsorption is commonly used in sepsis and septic shock to adsorb an excess of circulating pro-inflammatory mediators and ameliorate maladaptive hyperinflammation, and has also been applied in COVID-19 [4,5]. Recently, there has been considerable discussion and medical debate focused on the rationale of combining various EBP techniques in simultaneous or sequential modes in order to leverage their distinct or complementary properties for better clinical effect [6]. In line with this concept, in our cohort, we combined two haemoadsorption devices (CytoSorb^®^; CytoSorbents Europe GmbH, Berlin, Germany and Oxiris^®^; Baxter; Meyzieu; France within a single circuit, aiming to achieve a potentially stronger and faster effect.

The rationale for this approach was that, given the fulminant course of the disease, rapid and intensive intervention was required to counteract uncontrolled, overwhelming inflammation and interrupt the self-perpetuating inflammatory cascade. We therefore hypothesised that a multi-target strategy combining cytokine removal with endotoxin adsorption could improve prognosis. Up to 70% of secondary bacterial infections in COVID-19 are caused by Gram-negative bacteria [7], which release endotoxin (lipopolysaccharide, LPS), a potent driver of systemic inflammation [8]. High endotoxin levels have been detected in severely ill patients with COVID-19, particularly those admitted to the intensive care unit (ICU), together with high levels of circulating cytokines [9]. Accordingly, we used continuous renal replacement therapy (CRRT) with Oxiris^®^, with an additional cytokine adsorber—CytoSorb^®^—connected post-filter in the circuit. The therapeutic idea was to combine the large cytokine adsorption capacity of CytoSorb^®^ with the unique property of Oxiris^®^, which, in addition to modest cytokine removal, has the added ability to adsorb negatively charged endotoxins. We hypothesised that this strategy could contribute to more effective modulation of the dysregulated host response.

## 2. Methods

### 2.1. Aim

The objective of this single-centre retrospective study was to investigate the effect of EBP using a combination of an Oxiris^®^ haemodialysis filter set and a CytoSorb^®^ adsorber. The study population comprised critically ill patients with COVID-19 who developed secondary bacterial sepsis with persistent hemodynamic instability requiring vasopressor support.

### 2.2. Patients and Ethics

After approval from the Jagiellonian University Ethics Committee was obtained (approval number 1072.6120.188.2022; 12 October 2022), records of all patients hospitalised from October 2020 to March 2022 in the ‘COVID’ Intensive Care Unit of the University Hospital in Krakow were screened. Due to the retrospective design of the study, the need for informed consent was waived by the Jagiellonian University Ethics Committee.

The inclusion criteria were the following: confirmed COVID-19 infection; suspected or confirmed superimposed secondary infection of bacterial origin; need for vasopressors to maintain mean blood pressure (MAP) ≥ 65 mmHg despite adequate fluid therapy; indications for renal replacement therapy (RRT); and combined use of Oxiris^®^ haeamofilter with CytoSorb^®^ adsorber.

All patients were initially treated following the International Guidelines for Management of Sepsis and Septic Shock 2021 [10].

Microbiological cultures were taken, and broad-spectrum antibiotics were initiated immediately. A high-dose antimicrobial treatment regimen was used in all patients to account for potential drug removal or adsorption during extracorporeal therapy. In case of a PaO_2_/FiO_2_ ratio less than 150, prone positioning was used.

The study was carried out according to the STROBE (Strengthening the Reporting of Observational Studies in Epidemiology) guidelines [11] and was conducted in accordance with the Declaration of Helsinki.

### 2.3. Analysed Data and Scores

We retrospectively extracted clinical data from patient records. All data were collected and analysed anonymously.

Plasma concentrations of procalcitonin (PCT) and interleukin-6 (IL-6) levels, and leucocyte count were measured before the initiation of haemoadsorption (pre-haemoadsorption; pre-HA) and after the procedure (post-haemoadsorption; post-HA). The severity of organ dysfunction was evaluated with the Sequential Organ Failure Assessment (SOFA) at inclusion and before and after the haemoadsorption session. The PaO_2_/FiO_2_ ratio and vasopressor doses were recorded before and after the haemoadsorption session. To express the need for vasopressors, the noradrenaline equivalent dose (NED) was calculated based on the formula proposed by Goradia et al. [12]: NED = noradrenaline (µg/kg/min) + adrenaline (µg/kg/min) + vasopressin (IU/min) × 2.5.

### 2.4. Extracorporeal Treatment

Extracorporeal treatment consisted of continuous renal replacement therapy (CRRT) with the Oxiris^®^ haemofilter, with an additional cytokine adsorber—CytoSorb^®^—connected post-filter in the circuit. The rationale for this approach is that the CytoSorb^®^ cartridge is designed to adsorb and remove small- and medium-sized molecules ranging from 5 to 60 kDa, with a large adsorption surface of 45,000 m^2^ [13]. However, CytoSorb^®^ is not effective at adsorbing hydrophilic substances, including endotoxins, since they cannot diffuse to the adsorptive sites of the cartridge [14,15]. The Oxiris^®^ membrane (Baxter) is a heparin-coated haemofilter suitable for CRRT, with a smaller absorptive capacity compared with CytoSorb^®^. Its adsorptive spectrum covers cytokines of a molecular weight up to 35 kDa, and adsorption is based on hydrophilic interactions with the adsorptive surface of 17,500 m^2^. Due to an additional positively charged polyethyleneimine coating (PEI), Oxiris^®^ can also adsorb negatively charged endotoxins, which is not possible for CytoSorb^®^ [8,13]. Haemodiafiltration was started in patients with indications for renal replacement therapy, including severe acidosis, hyperkalaemia, persistent or progressive acute kidney injury (stage 2 or 3 according to KDIGO criteria), or pulmonary oedema/fluid overload not responding to diuretic treatment [16]. It was performed with the use of Prismaflex^®^ (GAMBRO Industries, Meyzieu, France) or PrismMax^®^ (Baxter; Meyzieu; France) Systems with regional citrate anticoagulation. The additional haemoadsorption with the CytoSorb^®^ adsorber was started when a rapid and substantial deterioration of the patient’s status was observed, expressed by an increase in the SOFA score of at least two points, not responding to initial treatment. The CytoSorb^®^ adsorber was connected post-haemofilter via a closed-loop circuit to the CRRT pump. The blood flow rate was established between 150 and 250 mL/min, and the dialysis dose varied from 20 to 35 mL/kg/h. Systemic and post-filter ionised calcium levels and acid-base balance were measured every 6 h to monitor and adjust haemodialysis settings. CytoSorb^®^ therapy was stopped after 24 h of cartridge usage unless further deterioration of haemodynamic status was observed. CRRT with Oxiris^®^ filter set was continued up to 72 h or longer if there were still indications for RRT. Subsequent CRRT, if indicated, was continued with the use of an Oxiris^®^ haemofilter or ST150 haemofilter (Baxter; Meyzieu; France), depending on the potential indications for further removal of cytokines (signs of infection, haemodynamic instability, or high inflammatory marker levels).

### 2.5. Statistical Analysis

The distribution of categorical variables was presented as numbers and percentages, numerically as means with standard deviations (SD) for normally distributed continuous variables, and as medians with interquartile ranges (IQR) for continuous variables with a skewed distribution. A Kolmogorov–Smirnov test was used to verify the normality of the data distribution. Comparisons were performed with Student’s *t*-test (for normally distributed variables) or a Mann–Whitney U test (for non-normally distributed variables). All statistical tests were two-sided, and a *p*-value ˂ 0.05 was considered statistically significant. An analysis of the required sample size was not performed due to the character of the study (proof-of-concept, pilot study). All analyses were performed in SPSS version 28.0 for Windows (SPSS, Chicago, IL, USA).

## 3. Results

### 3.1. Characteristics of the Study Population

From October 2020 to March 2022, 479 patients with COVID-19 infection were hospitalised in the ‘COVID-19′ Intensive Care Unit of the University Hospital in Krakow. Among these patients, 130 had indications for RRT and had haemodiafiltration started with the use of Oxiris^®^ haemofilter. In this group, we identified 12 patients who developed secondary sepsis of confirmed or suspected bacterial origin with persistent hemodynamic instability requiring vasopressor support, and who were treated with additional haemoadsorption with the use of CytoSorb^®^ adsorber and were included in the study.

**Figure 1:**
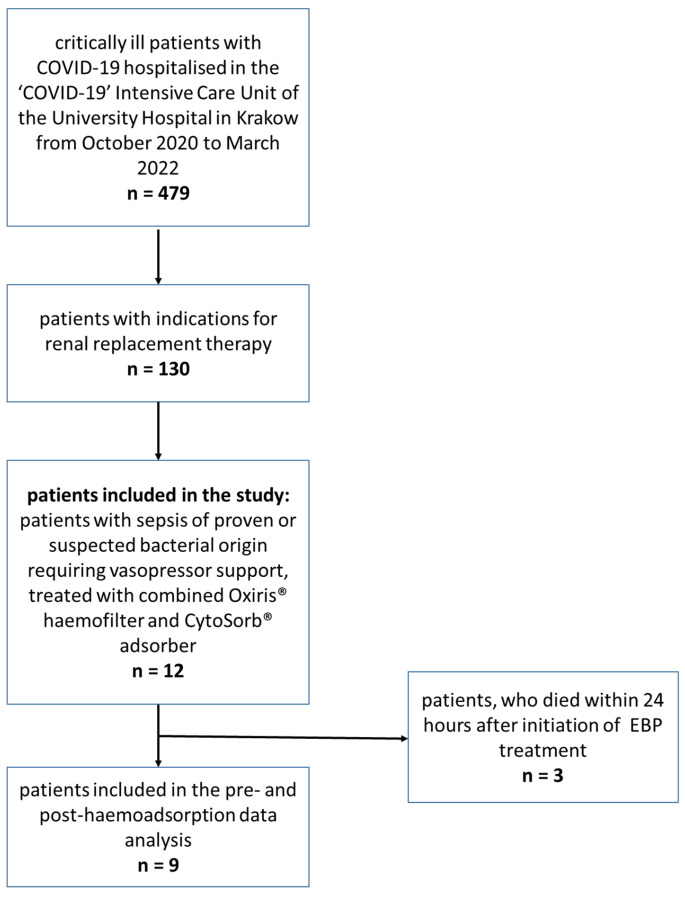
Patient flow diagram.

The patient flow diagram is presented in Figure 1. The demographic and clinical data of the included patients are presented in Table 1.

At the beginning of the EBP, all patients were on mechanical ventilation, and all required vasopressors to achieve a mean arterial pressure above 65 mmHg (median NED 0.5 ± 0.16 µg/kg/min). The mean SOFA score was 16.33 ± 1.37, the mean PaO_2_/FiO_2_ ratio was 99.5 ± 32.02, and the median procalcitonin and IL-6 levels were 10.8 ± 19.95 and 2417 ± 16,615, respectively. The blood cultures confirmed bacterial infection in all included patients; in 75% of them, it was caused by Gram-negative pathogens (Table 1).

### 3.2. Extracorporeal Treatment Characteristics

The CRRT with the use of the Oxiris^®^ filter set and CytoSorb^®^ adsorber therapy was started simultaneously in all patients. One CytoSorb^®^ cartridge was used for a maximum of 24 h, as indicated by the manufacturer. Most of the patients were treated with only one adsorber; In two patients, the adsorber had to be exchanged earlier due to clogging (one patient received two adsorbers, and one patient received three adsorbers). The mean total duration of CytoSorb^®^ therapy (including cartridge exchange) was 25.25 h (7 to 58 h). The Oxiris^®^ haemofilter was used for a maximum of 72 h, as indicated by the manufacturer; the mean duration of Oxiris^®^ therapy was 53.83 h (7 to 110 h); in most patients (nine patients), only one device was used, two patients received two devices, and one patient received three.

### 3.3. Outcomes

Pre-haemoadsorption (pre-HA) and post-haemoadsorption (post-HA) data were analysed in nine patients, who completed at least a 24 h course of treatment and for whom the results were available for comparison.

**Figure 2:**
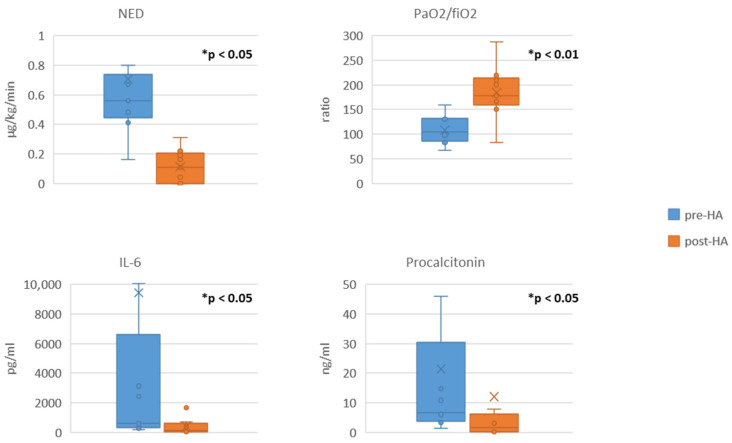
Comparison of pre-haemoadsorption (pre-HA) and post-haemoadsorption (post-HA) data. Data are presented in box-plots; data shown for nine patients who completed at least 24 h of treatment (*n* = 9) for each comparison; * indicates statistical significance; NED, noradrenaline equivalent dose; IL-6: interleukin-6.

We observed a statistically significant decrease in the SOFA score (Table 2). The vasopressor requirement (expressed as NED), PCT, and IL-6 level also decreased significantly; in fact, the medians decreased by 80.4%, 75.8%, and 81.7%, respectively (shown in Figure 2) The leucocyte count and lactate levels decreased, while the PaO_2_/FiO_2_ ratio increased (means increased by 41.5%) (shown in Table 2, Figure 2). A small drop in the haemoglobin levels and a moderate drop in the platelet count were observed during the treatment (Table 2). We did not observe any device-associated adverse events or technical problems associated with the circuit. Comparison of pre- and post-haemoadsorption data between survivors and non-survivors is provided in Supplementary Materials (Supplementary Figure S1).

Microbiological culture results confirmed bacterial co-infection in all of the patients.

The 28-day mortality in our study was 75%. In four out of the 12 included patients, the cause of death was associated with the progression of sepsis and haemodynamic instability, which led to the development of multiorgan failure. The reason for death for the other five patients was not associated with a progression of the treated sepsis. It occurred in the later course of ICU treatment (over 10 days after start of EBP) and was caused by other reasons, which included respiratory failure in two patients, cardiac arrest in one, and heart failure in the other two.

## 4. Discussion

In our study, the combined use of two haemoadsorption devices, Oxiris^®^ and CytoSorb^®^ cartridges, was associated with a decrease in vasopressor requirements and improvement in the SOFA score and PaO_2_/FiO_2_ ratio in critically ill patients with COVID-19, who developed secondary bacterial sepsis. Additionally, a substantial decrease in procalcitonin (PCT) and interleukin-6 (IL-6) levels and leucocyte count was observed.

Recently, a substantial number of clinical trials have emerged on the use of EBP techniques in sepsis and septic shock, including those involving patients with COVID-19 infection [15,17]. Some of them present very promising results, while others do not show superiority of EBP over conventional treatment or even show increased risk of mortality and serious complications [18,19,20]. A recent systematic review and meta-analysis by Steindl et al. suggests that the use of CytoSorb^®^ as an adjuvant therapy to the standard of care in patients with septic shock may be associated with a decrease in the mortality rate [21], and a meta-analysis by Wang et al. suggests a survival benefit with the adjuvant use of Oxiris^®^ in patients with sepsis undergoing CRRT [22]. However, other meta-analyses do not consistently demonstrate a clear benefit of haemoadsorption in sepsis and inflammation; Becker et al. did not show a survival benefit, although the population was not limited to patients with septic shock. Heymann et al., in a trial sequential meta-analysis for CytoSorb^®^, even warned that the therapy might increase mortality [23,24]. Nonetheless, it should be pointed out that these reports combine very heterogeneous studies in which EBP was used in various patients and settings, rarely in patients presenting symptoms of ‘cytokine storm’ in early sepsis. A lot of emphasis has been put lately on proper patient selection (the ones with a hyperinflammatory phenotype are most likely to benefit), early initiation of treatment to interrupt the self-perpetuating cascade, and limiting treatment duration to avoid immune over-depletion [19].

There is an increasing interest among researchers and clinicians in combining various EBP techniques with diverse and complementary properties to increase efficacy and adequacy of treatment. Premuzic et al. reported a positive effect of therapy with Oxiris^®^ or CytoSorb^®^ in combination with Seraph-100 Microbind Affinity Blood Filter in 68 critically ill COVID-19 patients with secondary bacterial co-infection [25], and Hui et al. reported sequential use of CytoSorb^®^ and Oxiris^®^ in a 14-year-old male with multisystem inflammatory syndrome after COVID-19 infection [26]. Nonetheless, simultaneous use of the two devices was reported previously only by Ferraro et al. in a single case report, in which a patient with severe endotoxin septic shock due to Neisseria meningitides was successfully treated with such EBP modality [27], and Wlochacz et al., who combined CytoSorb^®^ and Oxiris^®^ in a 67-year-old man with recurrent septic shock [28]. Thus, our study is the largest study available to date that provides data on the efficacy and safety of simultaneous use of Oxiris^®^ and CytoSorb^®^ in critically ill adult patients.

It should be noted that sepsis and septic shock in COVID-19 patients have a highly fulminant course and are associated with higher mortality and complication rates compared to patients without COVID-19 infection. Our previous clinical observations also confirm this fact, as we noticed that these patients present a particularly severe course of the disease and their status deteriorates within hours, leading in the majority of the cases to multiple organ failure and death. Little et al. reported 59.4% mortality in COVID-19 septic shock patients compared with 31.9% mortality in influenza septic shock patients [29]. Similar mortality rates are reported by Heubner et al. in patients with COVID-19 and sepsis, whereas Chen et al. reported a mortality rate as high as 97.7% in patients with COVID-19 and septic shock during the first wave of the pandemic [30]. These high mortality rates might be due to the fact that SARS-CoV-2 infection leads to severe immune disruption and immunodeficiency [2,31].

Endotoxin, more accurately referred to as lipopolysaccharide (LPS), is a part of the cell wall of Gram-negative bacteria, which plays a crucial role in protecting them from harsh environments, allowing colonisation of the human body, and evading the human immune system. It also has a key role in developing drug resistance [17,32]. In the human body, it is an alarm molecule that indicates microbial invasion. It has been acknowledged as one of the most potent mediators responsible for the development of severe sepsis, septic shock, and systemic inflammation. It triggers a rapid release of pro-inflammatory mediators and pro-coagulant factors [8,33]. High levels of endotoxins are associated with multiple organ failure and a high mortality rate in sepsis [34]. Elevated endotoxin levels have also been detected in COVID-19 patients in previous studies [13]. In our study, we enhanced the rate of cytokine removal by combining CytoSorb^®^ and Oxiris^®^, and, additionally, we removed endotoxin with the Oxiris^®^ haemofilter. We suspect that this EBP mode led to fast clearance of damage- and pathogen-associated molecular patterns (DAMPs and PAMPs) and thus attenuated and slowed the dynamics of inflammatory response in our patients and halted the self-reinforcing vicious cycle. We used CytoSorb^®^ for a single 24 h session unless further deterioration of haemodynamic status was observed. This approach is supported by medical evidence, in particular the two positive studies by Hawchar et al. [18] and Rugg et al. [20], in which CytoSorb^®^ was most often applied for a single 24 h session. Accordingly, we incorporated this regimen into our combined-mode configuration. It should also be noted that the adsorptive capacity of the Oxiris^®^ membrane is predominantly an early effect and is reported to saturate within approximately 24 h; thereafter, the oXiris^®^ cartridge functions as a conventional haemodialysis membrane [35]. It has to be emphasised, however, that cytokine and endotoxin removal should not be considered a standalone treatment of sepsis, but rather as an adjuvant therapy ‘buying time’ for other interventions to take effect, such as broad-spectrum antibiotics and source control. This can have even greater importance in patients with a dysregulated immune system, who are predisposed to hyperinflammatory conditions.

Our study shows a high level of reduction in pro-inflammatory markers (procalcitonin, interleukin-6 levels, and leucocyte count) and improvement in short-term clinical outcomes (PaO_2_/FiO_2_ ratio, SOFA score, and vasopressor requirement). However, despite observed improvements in short-term clinical markers, mortality remained very high (75%), which was in line with the severity of illness. The mortality rate among COVID-19 patients reported in other studies is also significant [28]. Our patient group had a particularly poor prognosis due to severe COVID-19, characterised by hemodynamic instability and secondary bacterial sepsis—both of which are strong additional risk factors for death. The basal SOFA score of the cohort was 16.33, which is associated with an expected mortality rate exceeding 95% [36]. Additionally, only four of the twelve included patients had a cause of death associated with progression of sepsis, while the other patients had different causes of death, which could not be directly influenced by EBP treatment.

We did not encounter technical problems associated with the circuit, including blood flow, clotting, or excessive device clogging. CytoSorb^®^ had to be changed within 24 h in two patients due to clogging, and Oxiris^®^ had to be changed within 72 h in one patient due to circuit clotting. During treatment, an 11.8% drop in haemoglobin level and a 27.5% drop in platelet count were observed (decrease from 269 ± 116 to 195 ± 82 × 10^6^/L before and post-haemoadsorption, respectively). It can be partially related to the blood purification technique; however, a drop in the platelet level is also a common observation in the course of sepsis and septic shock.

### 4.1. Future Directions

In recent years, increasing attention has been directed toward adjuvant therapies in the treatment of sepsis and septic shock. Although adherence to sepsis guidelines has significantly improved outcomes, mortality in high-risk patients remains unacceptably high, and additional therapeutic strategies are needed. Extracorporeal blood purification (EBP) represents one such option in patients with a hyper-inflammatory sub-phenotype, where overwhelming systemic inflammation contributes to organ failure and poor outcomes.

Future developments should focus on appropriate patient selection, precise sub-phenotype identification, and optimal timing of intervention. Combining complementary techniques—such as simultaneous endotoxin removal and high-volume cytokine adsorption, as applied in our study—may enhance the therapeutic effect through a multi-target approach. Better selection of patients based on biochemical signals, integrated with clinical status, is mandatory to allow for rapid bedside decision-making, since early implementation of a well-tailored intervention is critical.

Clearer guidelines are needed to support clinicians in identifying populations most likely to benefit. Further prospective, high-quality multicentre studies are warranted, with particular emphasis on rigorous selection of a sub-population of septic patients who may benefit.

Given the rapid innovation in sorbent and membrane technologies, EBP is evolving from a one-size-fits-all, single-device approach toward tailored, multi-hit strategies that simultaneously target key mediators and, potentially, pathogens, with the aim of maximising clinical benefit for patients.

### 4.2. Limitations

Our study has several serious limitations. The first limitation is a lack of a control group; second, the small number of patients included in the study; and third, the high mortality rate in the included group of patients. The above-mentioned reasons contribute to a high risk of possible bias, and thus, our results must be interpreted very carefully and have to be confirmed in further studies.

### 4.3. Conclusions

We present first early data on combined use of two EBP devices, Oxiris^®^ and CytoSorb^®^, within a single extracorporeal circuit for simultaneous cytokine and endotoxin removal, and our findings provide rationale for this approach. The results of our study in severe COVID-19 patients with secondary bacterial infection suggest a promising therapeutic option for other patients with virus-induced immune dysregulation who subsequently develop bacterial sepsis with an anticipated severe course. Our findings may serve as a guide for future research and provide directions for further development in this field. Future research on combining various EBP techniques to enhance clinical performance is of significant importance.

## Figures and Tables

**Table 1:** Characteristics of the study population.

Variable	Result (*n* = 12)
***Characteristics of the study population***	
Male gender, *n* (%)	6 (50)
Age, mean (SD)	50.3 (15.8)
BMI, mean (SD)	29.6 (5.3)
SOFA, mean (SD)	16.33 (1.37)
NED (µg/kg/min), median (IQR)	0.5 (0.16)
PaO_2_/FiO_2_, mean (SD)	99.5 (32.02)
IL-6 (pg/mL), median (IQR)	2417 (16,615)
Procalcitionin (ng/mL), median (IQR)	10.8 (19.95)
***Haemoadsorption***	
Duration of haemoadsorption with CytoSorb^®^ (h) [median (IQR)]	24.0 (6.0)
No. CytoSorb^®^ devices used	
- 1	10 (83%)
- 2	1 (8.5%)
- 3	1 (8.5%)
Duration of haemoadsorption with Oxiris^®^ (h) [mean (SD)]	53.8 (55.4)
No. of Oxiris^®^ devices used	
- 1	9 (75%)
- 2	2 (17%)
- 3	1 (8%)
Microbiology and Antibiotics *	
Gram-positive infection	3 (25%)
- Bloodstream infection	1 (8%)
- Respiratory tract infection	2 (17%)
Gram-negative infection	9 (75%)
- Bloodstream infection	2 (17%)
- Respiratory tract infection	8 (67%)
- Urinary tract infection	1 (8%)
Antibiotic treatment	
- Meropenem + Linezolid	5 (42%)
- Meropenem + Linezolid + Colistin	5 (42%)
- Meropenem + Linezolid + Ampicylin/Sulbactam	1 (8%)
- Cefepime + Ampicylin	1 (8%)
***Outcomes***	
Length of ICU stay (days), median (IQR)	12 (32)
Time of mechanical ventilation (days), mean (SD)	17 (17)
Duration of CRRT (hours), median (IQR)	48.0 (186.5)

BMI, body mass index; CRRT, continuous renal replacement therapy; ICU, intensive care unit; IQR, interquartile range; SD, standard deviation; SOFA, Sequential Organ Failure Assessment. * Percentages are calculated for the whole study population (*n* = 12). Totals may exceed 100% because some patients had more than one infection site. In three patients, multimicrobial infections were observed, and in four patients, MDR infections were identified.

**Table 2:** Comparison of pre-haemoadsorption (pre-HA) and post-haemoadsorption (post-HA) data.

	Pre-HA (*n* = 9)	Post-HA (*n* = 9)	Change (%)	*p*-Value	Statistical Test
SOFA, mean (SD)	16.3 (1.7)	15 (2.0)	−8.3%	*p* < 0.05	paired *t*-test
NED (µg/kg/min), median (IQR)	0.56 (0.29)	0.11 (0.21)	−80.4%	*p* < 0.05	Mann–Whitney U
PaO_2_/FiO_2_, mean (SD)	107.9 (28.8)	184.7 (55.3)	+41.5%	*p* < 0.01	paired *t*-test
IL-6 (pg/mL), median (IQR)	584 (6279)	107 (571)	−81.7%	*p* < 0.05	Mann–Whitney U
Procalcitonin (ng/mL), median (IQR)	6.5 (27)	1.6 (6)	−75.8%	*p* < 0.05	Mann–Whitney U
Leucocyte count (10^3^/mL), mean (SD)	36.0 (20.6)	20.91 (10.12)	−42.0%	*p* < 0.05	paired *t*-test
Lactate (mmol/L), mean (SD)	1.74 (0.74)	0.91 (0.26)	−47.7%	*p* < 0.05	paired *t*-test
Creatinine (µmol/L), mean (SD)	139 (97)	119 (80)	−14.4%	*p* = 0.2	paired *t*-test
PLT (10^6^/mL), mean (SD)	269 (116)	195 (82)	−27.5%	*p* < 0.05	paired *t*-test
Haemoglobin (g/dl), mean (SD)	10.7 (2.87)	9.5 (1.99)	−11.8%	*p* < 0.05	paired *t*-test
pH, mean (SD)	7.18 (0.12)	7.27 (0.10)	+1.2%	*p* = 0.6	paired *t*-test
BE (mEq/L), mean (SD)	−1.72 (4.26)	−3.8 (2.85)	−120%	*p* = 0.2	paired *t*-test

BE, base excess; PLT, platelet count; SD, standard deviation; SOFA, Sequential Organ Failure Assessment.

## Data Availability

The raw data supporting the conclusions of this article will be made available by the authors, without undue reservation.

## Supplementary Materials

The following supporting information can be downloaded at: https://www.mdpi.com/article/10.3390/jcm14196931/s1, Figure S1: Comparison of pre-haemoadsorption (pre-HA) and post-haemoadsorption (post-HA) data between survivors and non-survivors.
