# Supplementary material for: Is There a Rationale for Haemoadsorption with Combined Use of CytoSorb® and Oxiris® in Patients with Underlying Viral Infection and Secondary Bacterial Sepsis?
Source: J Clin Med. 2025 Sep 30;14(19):6931. doi: 10.3390/jcm14196931 (PMC12525037; doi:10.3390/jcm14196931)
Supplement: Supplementary file 1 [file jcm-14-06931-s001.zip › jcm-3813084-supplementary.pdf]

Supplementary file

Comparison of pre-haemoadsorption (pre-HA) and post-haemoadsorption (post-HA) data between survivors and non-survivors

Pre-haemoadsorption and post-haemoadsorption data were compared between survivors and non-survivors in 9 patients who completed at least 24 h of treatment. There were 3 patients in the survivor group and 6 patients in the non-survivor group. The analysis did not reveal any statistically significant differences between groups, most likely due to the small sample size. Therefore, no firm conclusions can be drawn from this analysis (Figure S1).

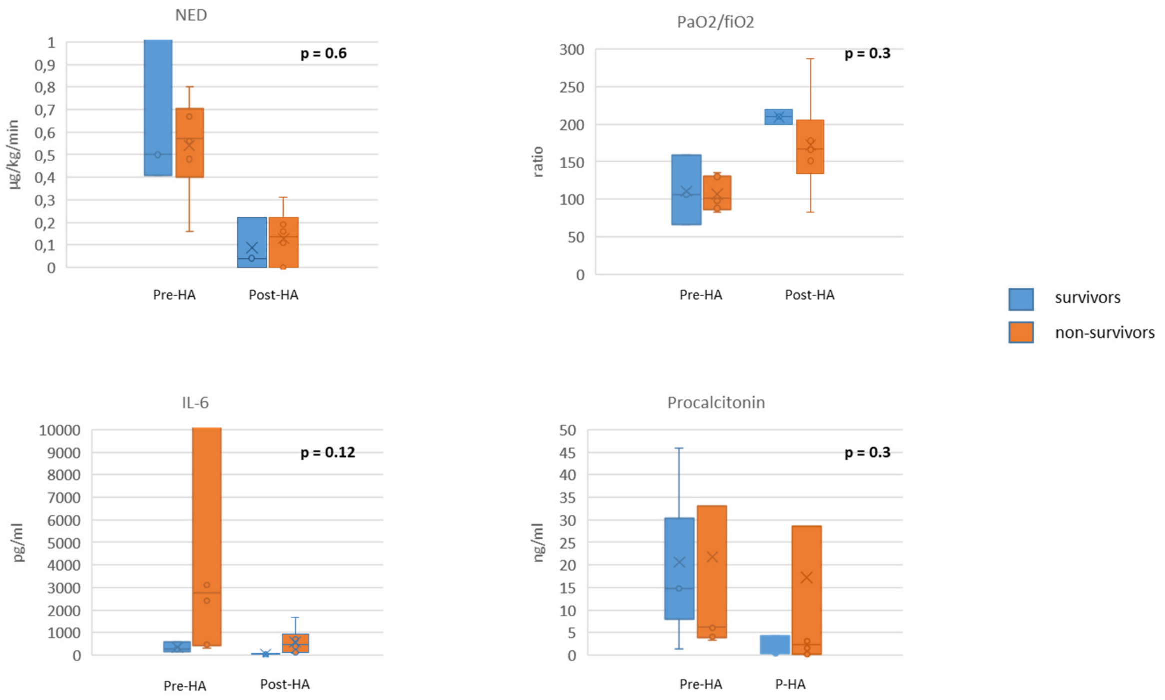

*Data are presented in box-plots; data shown for 9 patients who completed at least 24h of treatment for each comparison; *indicates statistical significance; NED, noradrenaline equivalent dose; IL-6: interleukin-6*

Figure S1. Comparison of pre-haemoadsorption (pre-HA) and post-haemoadsorption (post-HA) data between survivors and non-survivors.
